# SMA-13 Recycled Asphalt Mixtures with Flocculent Basalt Fiber: Experiment and Random Forest Analysis

**DOI:** 10.3390/ma19040649

**Published:** 2026-02-08

**Authors:** Yu Cai, Hong Sun, Zhipeng Tao, Kaimin Fu, Huajie Yin, Maomao Chen, Shenghan Zhuang, Jiaolong Ren

**Affiliations:** 1 Jiangxi Ganyue Expressway Co., Ltd., Nanchang 330025, China; caiyu_jx@126.com (Y.C.); chenmiaao@126.com (M.C.); 2 Jiangxi Provincial Transportation Consulting Co., Ltd., Nanchang 330038, China; 3 Jiangxi Provincial Communications Investment Maintenance Technology Group Co., Ltd., Nanchang 330213, China; taozpp@126.com; 4 Jiangxi Communications Investment Group Co., Ltd., Nanchang 330025, China; fukmin@126.com; 5 Jiangxi Provincial Transportation Construction Engineering Quality and Safety Assurance Center, Nanchang 330096, China; rrr_jx3657@126.com; 6 Department of Bridge Engineering, Southwest Jiaotong University, Chengdu 610031, China; shenghanzhuang@my.swjtu.edu.cn; 7 School of Civil Engineering and Geomatics, Shandong University of Technology, Zibo 255000, China

**Keywords:** SMA-13 recycled asphalt mixture, flocculent basalt fiber, road performance, fiber content, fiber diameter, fiber length, random forest

## Abstract

Flocculent basalt fiber (FBF), a natural fiber characterized by high strength, excellent toughness, and environmental friendliness, is an ideal additive for enhancing the road performance of recycled asphalt mixtures. However, existing research on FBF-reinforced recycled asphalt mixtures has largely been limited to single-factor analyses of FBF content, neglecting the synergistic effects of FBF size characteristics (diameter and length) and content. This critical gap restricts the accurate optimization of FBF parameters and the reliable application of FBF in recycled asphalt mixtures. Hence, this study investigates the combined effects of FBF diameter, length, and content on the optimal asphalt–aggregate ratio, mechanical properties, high-temperature rutting resistance, low-temperature cracking resistance, and water stability of SMA-13 recycled asphalt mixtures. A random forest approach is adopted to quantify the relative importance of FBF diameter, length, and content on the optimal asphalt–aggregate ratio and various road performance indexes. The results show that the optimal asphalt–aggregate ratio and road performance indexes increase significantly with increasing FBF content and length but decrease with increasing FBF diameter, with minimal variation in replicate tests. However, when the fiber content surpasses 0.4%, a deterioration in performance occurs. Fiber content has the most significant impact on the optimal asphalt–aggregate ratio and overall road performance, followed by diameter and then length. The optimal fiber content is identified as 0.4% for fibers with a diameter of 6 µm (regardless of fiber length in this study) and 0.3% for fibers with a diameter of 3 µm and a length of 4 mm. These findings provide precise parameter guidance for engineering applications of FBF in SMA-13 recycled asphalt mixtures, thereby promoting the sustainable utilization of recycled materials.

## 1. Introduction

Basalt fiber, a natural fiber obtained from basalt rock through specialized processing techniques, exhibits several beneficial properties, including low density, high tensile strength, notable toughness, and abundant raw material availability [1]. The production of basalt fiber is characterized by low carbon emissions and minimal environmental pollution, rendering it an environmentally sustainable material that aligns with China’s green development objectives [2,3]. As a result, basalt fiber has experienced increased utilization in civil engineering applications in recent years. Basalt fibers are typically classified into two categories: chopped bundle basalt fibers (CBFs) and flocculent basalt fibers (FBFs) [4]. CBFs are manufactured by melting basalt rock at elevated temperatures, followed by fiber drawing and treatment with lipophilic impregnating agents to form fiber bundles [5]. Conversely, FBFs are produced through high-temperature melting of basalt rock, succeeded by high-speed centrifugal spinning, purification, and treatment with a silane-coupling agent [6]. This agent functions as a cationic impregnating substance that adsorbs onto the fibers via its cationic moiety and establishes covalent bonds with the fiber surface through hydrolysis and condensation reactions of siloxane. This mechanism effectively links the inorganic fibers with organic asphalt, thereby enhancing interfacial adhesion.

Currently, the CBFs are primarily utilized as short fibers in civil engineering materials, including asphalt mixtures and cement concrete. This approach represents the main application method of basalt fibers within the civil engineering field and has proven to be highly effective [7,8,9,10]. Nevertheless, despite the enhanced mechanical properties of CBFs relative to FBFs, their dispersion within high-viscosity asphalt mixtures remains challenging, thereby complicating construction procedures [11]. Additionally, the bundling manufacturing process results in increased production costs, which restricts their widespread use in cost-sensitive pavement engineering applications [12].

In contrast to engineering structures such as bridges and tunnels, which require basalt fibers with exceptionally high structural strength, asphalt pavements do not impose stringent mechanical property requirements on the fibers used. Instead, emphasis is placed on achieving uniform fiber dispersion, facilitating ease of construction, and ensuring cost-effectiveness [13]. As a result, the FBFs are considered particularly well-suited for incorporation into asphalt mixtures, leading to extensive research aimed at exploring their potential applications. Zhao et al. [14] examined the influence of CBF content on the performance of AC-13 asphalt mixtures, thereby confirming the feasibility of integrating basalt fibers into asphalt composites. However, their study did not address the impact of FBF on the road performance of asphalt mixtures. Wu et al. [15] and Cai et al. [16] modified SMA-13 asphalt mixtures using four different fiber types and demonstrated that FBF provided more comprehensive performance improvements than other fibers, particularly with respect to high-temperature stability. Nevertheless, these studies considered only a single variable—FBF content—leaving a gap in the systematic evaluation of FBF size parameters (length and diameter) and their interactive effects with fiber content. Wang et al. [17], Yang et al. [18], and Xing et al. [19] investigated warm-mix recycled asphalt mixtures modified with both CBF and FBF at varying contents, focusing primarily on their cracking behavior. These studies concluded that the inclusion of FBF significantly enhanced the cracking resistance of recycled asphalt mixtures compared to those containing CBF. However, a detailed analysis of the size characteristics of FBF was not provided in these investigations.

Previous research has primarily focused on examining the influence of individual parameters, such as fiber type or fiber content, on the road performance of various asphalt mixtures [20,21]. Nonetheless, there remains a notable gap in comprehensive studies that analyze the combined effects of three key factors—fiber diameter, length, and content—within recycled asphalt mixtures (RAMs) containing the FBFs [22]. This gap impedes the broader application and practical utilization of FBF in pavement engineering. Furthermore, in the context of China’s commitments to reach a peak in carbon dioxide emissions by 2030 and achieve carbon neutrality by 2060, the engineering significance of RAMs has attracted growing academic attention. Typically, RAMs exhibit relatively inferior performance and stability compared to conventional asphalt mixtures, often necessitating reinforcement through fiber addition [23,24].

Accordingly, the present study aims to investigate the synergistic effects of FBF size parameters (diameter and length) and fiber content on the road performance of RAMs. Previous investigations into fiber effects have largely employed traditional multiple linear regression models, which are limited in their capacity to capture complex nonlinear interactions [25]. In contrast, machine learning techniques have shown enhanced predictive accuracy and generalizability in modeling the behavior of construction materials [26].

This research evaluates the impact of FBF size characteristics on the optimal asphalt–aggregate ratio (OAR), mechanical properties, high-temperature stability, low-temperature crack resistance, and water stability of SMA-13 recycled asphalt mixtures (RAM-SMA-13) across varying fiber contents. Additionally, it integrates multifactorial experimental designs with a random forest (RF) modeling approach to quantitatively assess the interactive effects of FBF size and content on RAM-SMA-13 properties. The results offer a foundational framework to inform the compositional design of FBF-reinforced recycled asphalt mixtures.

## 2. Materials and Test Preparation

### 2.1. Materials

The SBS-modified asphalt, together with the coarse aggregate, fine aggregate, and filler, were produced and supplied by Jiangxi Ganuo Expressway Co., Ltd., Nanchang, China. The technical properties of these materials are presented in Table 1, Table 2, Table 3 and Table 4, respectively, and conform to the requirements specified in the Chinese standard “Technical Specification for Construction of Highway Asphalt Pavements (JTG F40-2004).” [27]. The SBS content in the modified asphalt is maintained at a mass fraction of 4.5%, with a number average molecular weight of 120,000 g/mol. The PG grade of the SBS-modified asphalt is classified as PG 76-22. The coarse aggregate is basalt, the fine aggregate is sourced from river sand, and the filler consists of limestone powder.

Recycled aggregates derived from the screening of milled asphalt pavement materials are produced in two distinct size fractions: 0–8 mm and 8–12 mm. The technical characteristics of these aggregates are detailed in Table 5.

The FBFs utilized in this research were procured from Hangzhou Jialu Transportation Technology Co., Ltd., Hangzhou, China. These fibers are available in three distinct specifications, where the first parameter denotes the diameter and the second corresponds to the length: d—6 μm × L—4 mm, d—3 μm × L—4 mm, and d—6 μm × L—2 mm. The technical characteristics of the fibers are presented in Table 6.

### 2.2. Instruments and Equipment

Mechanical property assessments of asphalt mixtures are conducted utilizing the LHPL-6 comprehensive performance testing apparatus. Rutting resistance is evaluated through the application of the LHCX-1 asphalt mixture rutting tester (Beijing Zhongjian Luye Instrument Equipment Co., Ltd., Beijing, China). Furthermore, low-temperature beam bending tests at −10 °C, along with freeze–thaw splitting tests, are performed using the LHZH-6 low-temperature comprehensive performance testing device for asphalt mixtures.

### 2.3. Sample Preparation

Table 7 presents the gradation range for RAM-SMA-13. In each aggregate specification, 60% of the total mass is replaced with recycled aggregates. Sample preparation follows the standardized protocols outlined in the Chinese standard “Standard Test Methods of Asphalt and Asphalt Mixture for Highway Engineering (JTG 3410-2025)” [28].

Due to the high specific surface area of the FBF and its propensity to agglomerate during mixing—factors that negatively impact the road performance of the mixture—the following preparation procedure was adopted in this study:

Step 1: The FBF was dried in an oven at 60 °C for 2 h to reduce its moisture content to below 0.5%. The dried fibers were then subdivided into smaller portions to minimize agglomeration caused by batch feeding.

Step 2: The recycled and virgin aggregates were thoroughly mixed and preheated to 180 °C.

Step 3: A total of 70% of the preheated aggregates were added to the mixer and dry-mixed for 10 s to form the aggregate base mixture.

Step 4: While the mixer remained in operation, the FBF was gradually introduced into the aggregate base mixture, followed by the addition of the remaining preheated aggregates.

Step 5: The mixture was considered homogeneously combined after continuous stirring for 120 s.

### 2.4. Performance Testing

Mechanical properties are evaluated through compressive strength and splitting strength tests. High-temperature performance is assessed using the rutting test, while low-temperature performance is measured by the bending test conducted at −10 °C. Water stability is examined via the freeze–thaw splitting test. All experimental procedures adhere to the Chinese standard “Standard Test Methods of Asphalt and Asphalt Mixtures for Highway Engineering (JTG 3410-2025).” To ensure the reliability of the results, five samples are used for determining the optimum asphalt content (OAR), three samples for the rutting test, and six samples for the strength, bending, and freeze–thaw splitting tests.

## 3. Influence of Flocculent Basalt Fibers on the OAR

### 3.1. Fiber Diameter

**Figure 1:**
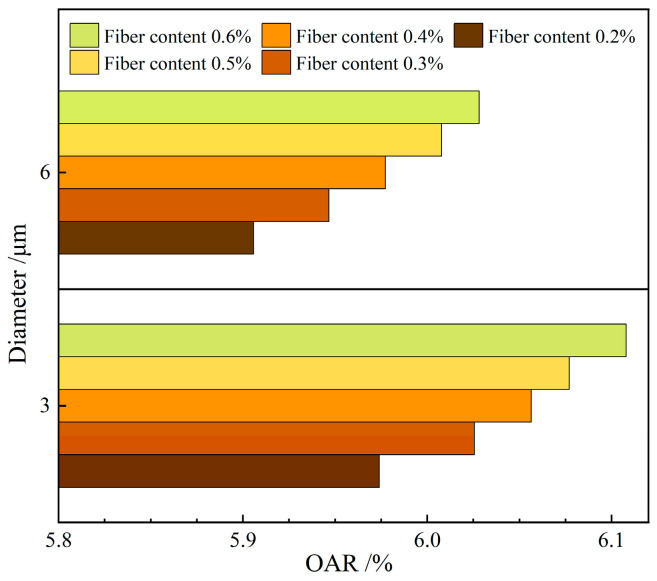
Effect of fiber diameter on the OAR.

Figure 1 illustrates the effect of varying diameters of the FBF on the OAR in RAM-SMA-13.

As illustrated in Figure 1, the OAR for fibers with a diameter of 3 μm surpasses that of fibers with a diameter of 6 μm, demonstrating an increase of approximately 1.26%. At the macroscopic level, a reduction in fiber diameter results in an augmented specific surface area, which in turn heightens the demand for asphalt adsorption. On a microscopic scale, the fibers affect the micro-mechanical behavior of the RAM-SMA-13 through adsorption processes. The increased specific surface area offers a greater number of contact points for asphalt, thereby enhancing the adsorption capacity [29,30]. As a result, the amount of structural asphalt adhering to the fiber surfaces rises, which restricts its effectiveness as a binder within the mixture and consequently leads to a gradual increase in the overall asphalt content. Moreover, in experimental investigations aimed at determining the OAR for basalt fiber-reinforced asphalt mixtures, Kou et al. utilized basalt fibers of three distinct diameters and found that smaller fiber diameters were associated with higher OAR values [31]. These results are consistent with previous studies, thereby supporting the validity of the proposed mechanistic explanation.

### 3.2. Fiber Length

**Figure 2:**
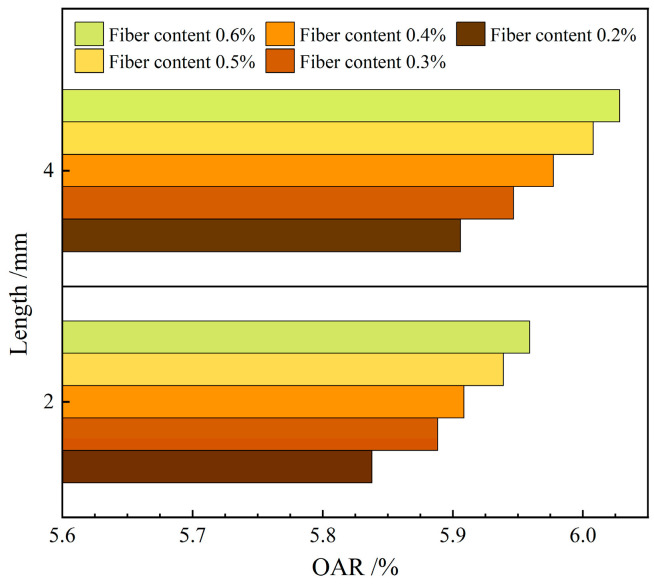
Effect of fiber length on the OAR.

Figure 2 illustrates the effect of varying lengths of the FBF on the OAR in RAM-SMA-13.

As depicted in Figure 2, when the FBF length is extended to 4 mm, the OAR increases by approximately 1.13% compared to that observed with a fiber length of 2 mm. Incorporating longer FBFs into RAM-SMA-13 necessitates a higher asphalt content to ensure sufficient fiber coating. This increased asphalt content improves bonding efficiency and connectivity, thereby promoting the development of a spatial network structure within the RAM-SMA-13. Lou et al. examined the OAR in asphalt mixtures with different maximum particle sizes and found that the optimal asphalt content tends to increase in parallel with fiber length [32]. Together, these findings, in conjunction with previous studies, elucidate the fundamental mechanism by which fiber length affects the OAR.

### 3.3. Fiber Content

**Figure 3:**
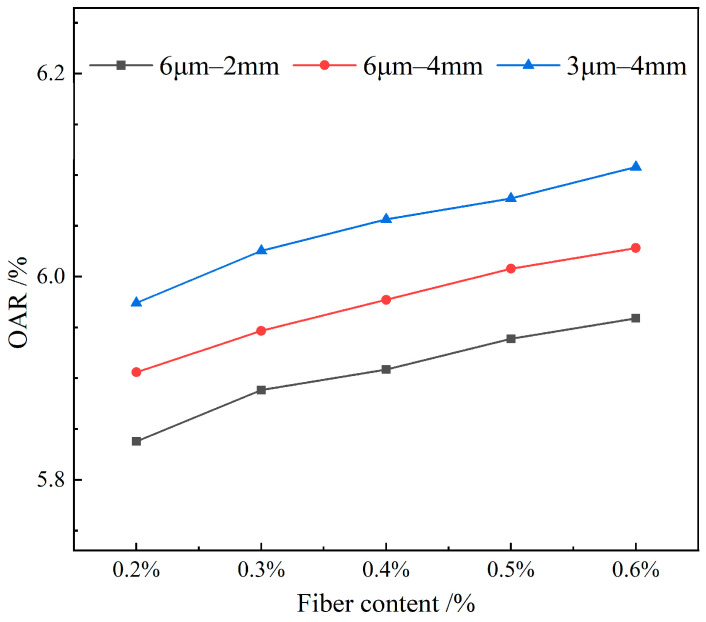
Effect of fiber content on the OAR.

Figure 3 illustrates the effect of varying contents of the FBF on the OAR in RAM-SMA-13.

As depicted in Figure 3, the OAR of RAM-SMA-13 demonstrates an increasing trend with the addition of FBF. The maximum observed increment for a specific fiber specification reaches 2.24%. This behavior can be attributed to the inherent asphalt absorption properties of the FBF. With an increase in FBF content, the total surface area of fibers within the RAM-SMA-13 correspondingly expands, thereby complicating their uniform dispersion. Consequently, a greater quantity of asphalt is required to sufficiently coat the fibers, leading to an elevated OAR. This adsorption phenomenon reduces the proportion of free asphalt to some extent while increasing the amount of structural asphalt, which in turn enhances the overall performance of RAM-SMA-13. Prior research has demonstrated that increasing basalt fiber content from 0% to 0.5% significantly raises the viscosity of asphalt [33]. Furthermore, the augmentation of fiber content affects the mixture’s performance by increasing the viscosity of the asphalt mortar [34]. The foregoing analysis, corroborated by existing studies, indicates that the substantial absorption of asphalt by FBF decreases the proportion of free asphalt, with fiber content showing a positive correlation with asphalt demand.

## 4. The Influence of Flocculent Basalt Fibers on the Road Performance

### 4.1. The Influence of Fiber Diameter

#### 4.1.1. Mechanical Properties

**Figure 4:**
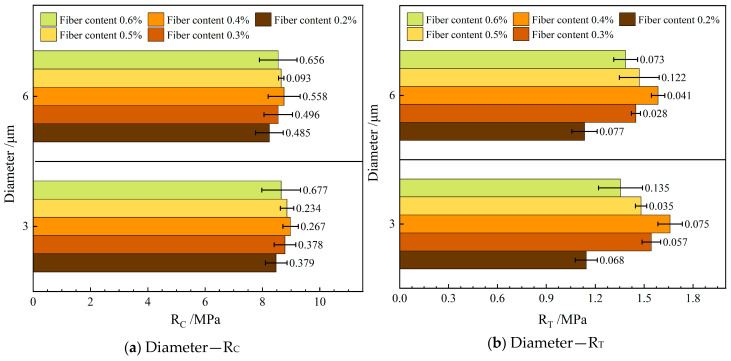
Effect of fiber diameter on mechanical properties.

Figure 4 depicts the influence of varying the diameter of the FBF on the mechanical properties in RAM-SMA-13. In this figure, R_C_ represents the compressive strength, and R_T_ denotes the splitting strength.

As illustrated in Figure 4, the mechanical properties of RAM-SMA-13 reinforced with d—3 μm FBF surpass those of the mixture containing d—6 μm FBF. Specifically, the average compressive strength of the mixture with d—3 μm FBF is 2.4% greater than that of the mixture with d—6 μm FBF, while the average splitting tensile strength shows an increase of 4.8%. The finer FBFs exhibit enhanced dispersion and uniformity within the mixture, along with a larger specific surface area, which promotes stronger adhesion to the asphalt binder and consequently improves the bonding strength of RAM-SMA-13. Furthermore, the incorporation of FBF facilitates a more homogeneous distribution of the three primary components—aggregate, asphalt, and fibers—thereby enabling more effective load transfer and enhancing the overall mechanical performance.

Concerning the improvement in splitting tensile strength, the study by Kou et al. demonstrated that 7 μm FBF increased the splitting tensile strength, compressive strength, and dynamic modulus of the asphalt mixture by 5.03%, 3.11%, and 4.08%, respectively, compared to 16 μm FBF [31]. This evidence further corroborates the superior performance associated with smaller diameter basalt fibers. The underlying mechanism aligns with findings from previous research, and the results of the present study confirm the applicability of this mechanism within the context of RAM.

#### 4.1.2. High-Temperature Stability

**Figure 5:**
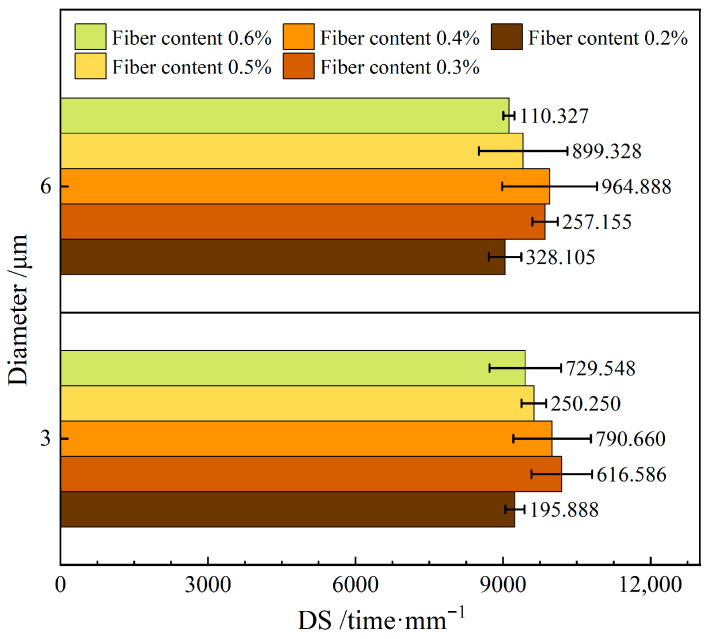
Effect of fiber diameter on high-temperature stability.

Figure 5 depicts the influence of varying the diameter of the FBF on the high-temperature stability in RAM-SMA-13. In this figure, DS denotes dynamic stability.

Figure 5 demonstrates that the dynamic stability of RAM-SMA-13 containing d—3 μm FBF is 2.5% higher than that of the composite with d—6 μm FBF. The decrease in fiber diameter leads to an increased fiber count and a greater specific surface area within the mixture. This alteration substantially enhances the asphalt adsorption capacity, facilitates the formation of structural asphalt, and reduces the proportion of free asphalt. Additionally, fibers with smaller diameters establish a denser spatial network within the composite, restricting the mobility of free asphalt at elevated temperatures and thereby improving the high-temperature stability of RMA-SMA-13.

Previous research has indicated that mixtures incorporating fibers of smaller diameters exhibit superior rutting resistance and, to some extent, enhanced high-temperature stability [31]. However, it is crucial to recognize that complex environmental factors may affect fiber performance. Xu et al. investigated waste crushed basalt fibers and found that fibers of varying diameters interact synergistically to influence the multi-temperature domain performance of asphalt mixtures, implying that the optimal fiber diameter is not necessarily the smallest [35]. This finding is consistent with the theoretical analysis presented here, suggesting that although finer fibers generally improve high-temperature stability, careful selection of an appropriate diameter range is essential.

#### 4.1.3. Low-Temperature Crack Resistance

**Figure 6:**
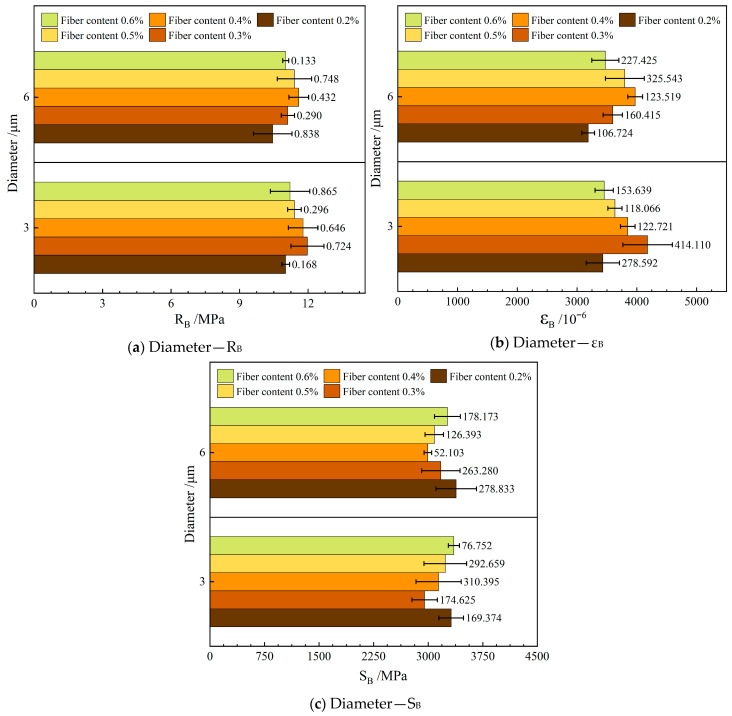
Effect of fiber diameter on low-temperature crack resistance.

Figure 6 depicts the influence of varying the diameter of the FBF on the low-temperature crack resistance in RAM-SMA-13. In this figure, R_B_ denotes the flexural tensile strength, ε_B_ represents the flexural tensile strain, and S_B_ corresponds to the flexural tensile modulus.

As illustrated in Figure 6, the RAM-SMA-13 reinforced with d—3 μm FBF exhibits markedly improved resistance to low-temperature cracking compared to the mixture reinforced with d—6 μm FBF. Specifically, relative to the d—6 μm FBF-reinforced mixture, the average flexural tensile strength of the d—3 μm FBF-reinforced mixture increased by 3.3%, the average flexural tensile strain improved by 4.8%, and the average flexural modulus decreased by 1.6%. At lower temperatures, the stiffness modulus of RAM-SMA-13 significantly decreases, which correlates with diminished flexibility and increased brittleness. The FBF possesses intrinsic toughness that effectively counteracts the brittleness of the composite material under these conditions. Additionally, the enhanced elastic modulus and tensile strength of the FBF contribute to an increased capacity for absorbing damage energy during crack propagation under applied loads, thereby augmenting the low-temperature crack resistance of RAM-SMA-13.

Previous studies have investigated the effects of larger fiber diameters, specifically 16 μm and 25 μm, on the low-temperature performance of SMA-13 mixtures. These studies reported no significant differences in performance between mixtures reinforced with these two fiber sizes across various temperatures and loading durations [30]. This finding contrasts with the results of the present study. It is hypothesized that the fiber diameters examined herein are substantially smaller than 16 μm, and the considerable size disparity between fibers leads to reduced flexibility and increased stiffness. This explains why fibers with diameters around 16 μm do not demonstrate notable advantages under low-temperature conditions.

These findings are consistent with the current study’s analysis, indicating that finer fibers primarily enhance low-temperature crack resistance through mechanisms of energy absorption. Moreover, this investigation provides insight into the microscopic mechanisms by which fiber diameter influences the low-temperature performance of asphalt mixtures.

#### 4.1.4. Water Stability

**Figure 7:**
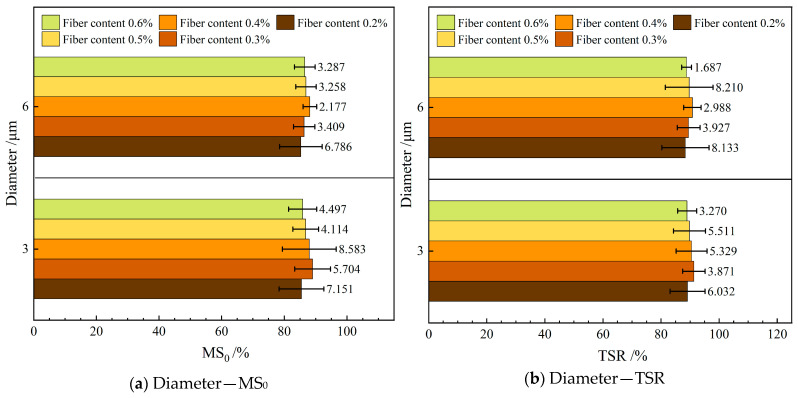
Effect of fiber diameter on water stability.

Figure 7 depicts the influence of varying the diameter of the FBF on water stability in RAM-SAM-13. In this figure, MS_0_ represents the residual stability, while TSR denotes the freeze–thaw splitting strength ratio.

As illustrated in Figure 7, the incorporation of d—3 μm FBF reinforcement markedly enhances the water stability of RAM-SMA-13 compared to reinforcement with d—6 μm FBF. Specifically, the mixture containing d—3 μm FBF demonstrates residual stability and freeze–thaw splitting strength values that surpass those of the d—6 μm FBF-reinforced mixture by 0.8% and 0.4%, respectively. This enhancement is primarily attributed to the increased specific surface area of the finer fibers, which augments their capacity for asphalt adsorption. As a result, the aggregates are more effectively encapsulated by the asphalt binder, leading to the development of a more stable asphalt film that significantly impedes water infiltration along the aggregate–asphalt interface. This mechanism delays the debonding process and reduces water penetration. Nonetheless, the fiber diameter has a limited effect on the internal bonding strength of the composite, resulting in only a slight improvement in its overall water stability.

### 4.2. The Influence of Fiber Length

#### 4.2.1. Mechanical Properties

**Figure 8:**
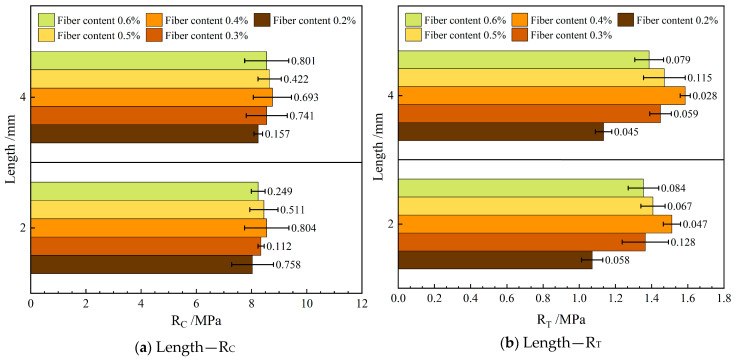
Effect of fiber length on mechanical properties.

Figure 8 shows the influence of varying the length of the FBF on the mechanical properties in RAM-SMA-13.

As illustrated in Figure 8, the RAM-SMA-13 reinforced with 4 mm FBF exhibits superior performance in both compressive strength and crack resistance compared to the mixture containing 2 mm FBF. Specifically, the compressive strength of the 4 mm FBF-reinforced mixture increases on average by 2.7%, while its crack resistance improves by 4.8% relative to the 2 mm FBF counterpart. These enhancements in mechanical properties are attributed to the longer fiber length, which facilitates the development of an interwoven fiber network within RAM-SMA-13, thereby improving the composite’s internal bonding. Furthermore, the concept of critical fiber length is relevant; fibers exceeding this threshold can sustain sufficient stress to fracture rather than being pulled out, thus contributing to the material’s resistance to failure. It is hypothesized in this study that the 4 mm fibers have reached or surpassed this critical length, which accounts for the significant performance gains observed in the 4 mm FBF-reinforced RAM-SMA-13 compared to the 2 mm variant.

Additionally, previous studies have demonstrated a positive relationship between basalt fiber length and improvements in flexural strength and crack resistance in AC-13 asphalt mixtures, lending support to analogous conclusions for RAM-SMA-13 [36]. This evidence reinforces the current study’s finding that longer fibers have a more substantial beneficial impact on the macroscopic mechanical properties of RAM.

#### 4.2.2. High-Temperature Stability

**Figure 9:**
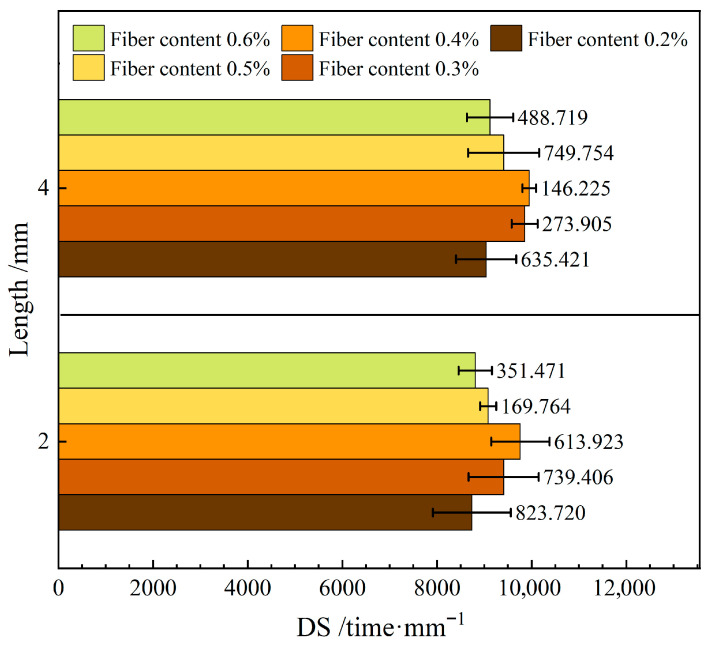
Effect of fiber length on high-temperature stability.

Figure 9 shows the influence of varying the length of the FBF on high-temperature stability in RAM-SMA-13.

As illustrated in Figure 9, the dynamic stability of RAM-SMA-13 containing fibers of a length of 4 mm (FBF) exceeds that of the mixture with 2 mm fibers by 4.1%. The increased fiber length promotes the development of a network structure within the composite, which effectively limits lateral displacement at elevated temperatures, thereby improving its high-temperature stability. In contrast, shorter fibers demonstrate reduced connectivity, leading to diminished stability of the mixture under high-temperature conditions.

Lou et al. [32] identified an optimal fiber length for asphalt mixtures with different nominal maximum particle sizes that maximizes high-temperature performance. As fiber length increases, performance improvements tend to plateau, and certain performance indicators may even decline. This behavior is primarily attributed to the dispersion characteristics of fibers within the mixture: although longer fibers theoretically enhance bridging and network formation, excessively long fibers are prone to entanglement and agglomeration during mixing. Such aggregation results in uneven fiber distribution and the formation of structurally weak regions, thereby reducing their reinforcing efficacy [37]. Collectively, these findings clarify the mechanistic relationship between FBF length and the high-temperature stability of RAM-SMA-13.

#### 4.2.3. Low-Temperature Crack Resistance

**Figure 10:**
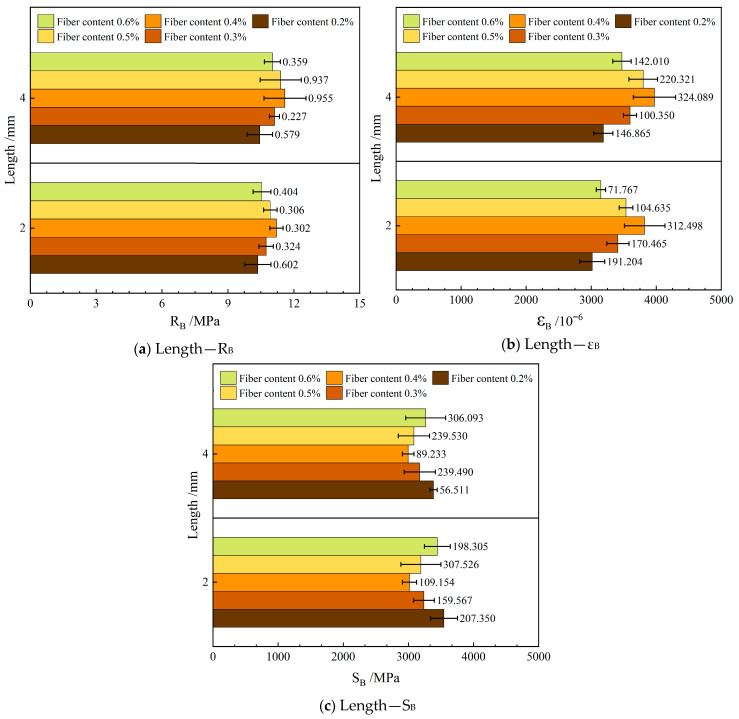
Effect of fiber length on low-temperature crack resistance.

Figure 10 shows the influence of varying the length of the FBF on the low-temperature crack resistance in RAM-SMA-13.

As illustrated in Figure 10, the crack resistance of RAM-SMA-13 reinforced with 4 mm long FBF surpasses that of the composite reinforced solely with 2 mm long FBF under low-temperature conditions. Specifically, relative to the 2 mm FBF variant, the mixture containing 4 mm FBF exhibits a 3.9% increase in bending tensile strength, a 6.4% enhancement in bending tensile strain, and a 2.4% reduction in bending modulus. The extended fiber length contributes to greater mixture flexibility, which facilitates effective crack bridging, enhances deformation energy absorption, and delays crack propagation during the initial stages. Collectively, these factors improve the crack resistance of RAM-SMA-13 at low temperatures.

During the beam bending test, the interlaced FBF imparts increased toughness to the specimen under shear stress. The effective length of the 4 mm fibers within the mixture exceeds that of the 2 mm fibers, approaching the critical fiber length, thereby improving stress transfer efficiency and increasing load-bearing capacity. These mechanisms collectively elucidate the impact of FBF length on the performance of RAMs at low temperatures.

#### 4.2.4. Water Stability

**Figure 11:**
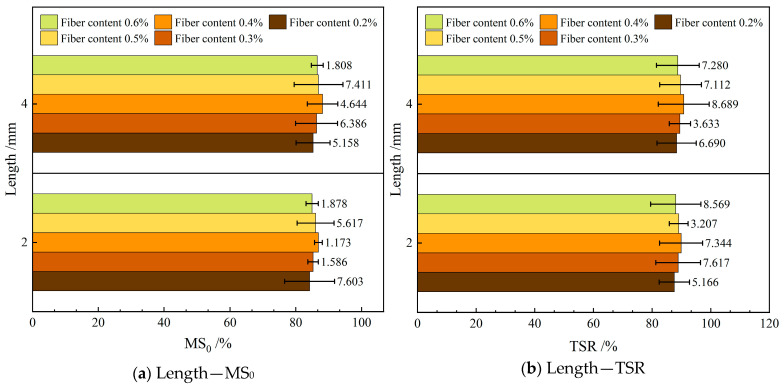
Effect of fiber diameter on water stability.

Figure 11 shows the influence of varying the length of the FBF on water stability in RAM-SMA-13.

As depicted in Figure 11, the water stability of RAM-SMA-13 incorporating 4 mm length FBF surpasses that of the composite containing 2 mm length FBF. Specifically, the residual stability of the former exhibits an increase of 1.0%, while the freeze–thaw splitting strength ratio improves by 0.7%. The longer fiber length facilitates enhanced absorption of free asphalt, leading to the development of an asphalt film on the aggregate surface. This film functions as a protective barrier against water penetration, thereby improving the water resistance characteristics of RAM-SMA-13.

### 4.3. The Influence of Fiber Content

#### 4.3.1. Mechanical Properties

**Figure 12:**
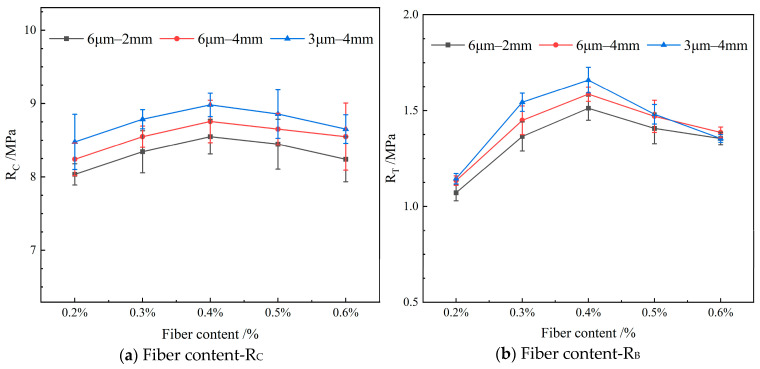
Effect of fiber content on mechanical properties.

Figure 12 depicts the influence of varying the content of FBF on the mechanical properties in RAM-SMA-13.

As depicted in Figure 12, the mechanical properties of RAM-SMA-13 initially improve with increasing fiber content, reaching an optimum at 0.4%, beyond which a decline is observed. This behavior can be explained by the fiber’s ability to effectively distribute applied stresses, mitigate stress concentrations, and absorb substantial amounts of asphalt. Such interactions facilitate the development of a distinct asphalt layer of defined thickness, wherein the structural asphalt encapsulates the fibers on the aggregate surfaces. This encapsulation enhances aggregate cohesion, thereby augmenting the mechanical performance of RAM-SMA-13. However, when the fiber content exceeds the optimal level, the absorption capacity of the fibers becomes saturated, resulting in a decrease in their relative effectiveness.

In comparison to the results reported by Wu et al. [36], where the optimal fiber content for the AC-13 mixture was determined to be 0.3%, the present study identifies 0.4% as the optimal fiber content for RAM-SMA-13, underscoring the impact of gradation differences. RAM-SMA-13 is characterized by an intermittent grading skeleton-dense structure, with its strength predominantly reliant on the compaction of coarse aggregates and the bonding provided by asphalt. At a fiber content of 0.4%, a stable three-dimensional fiber network is established, which compensates for the inherent interfacial weaknesses of recycled materials. In contrast, the AC-13 mixture exhibits a continuous grading suspended-dense structure, where strength primarily arises from asphalt–aggregate bonding without substantial skeletal support. In this context, a fiber content of 0.3% optimally reinforces bonding, whereas excessive fiber addition tends to induce agglomeration, thereby reducing resistance to bending deformation.

Overall, the analysis highlights the critical role of fiber content in influencing the macroscopic properties of RAM-SMA-13.

#### 4.3.2. High-Temperature Stability

**Figure 13:**
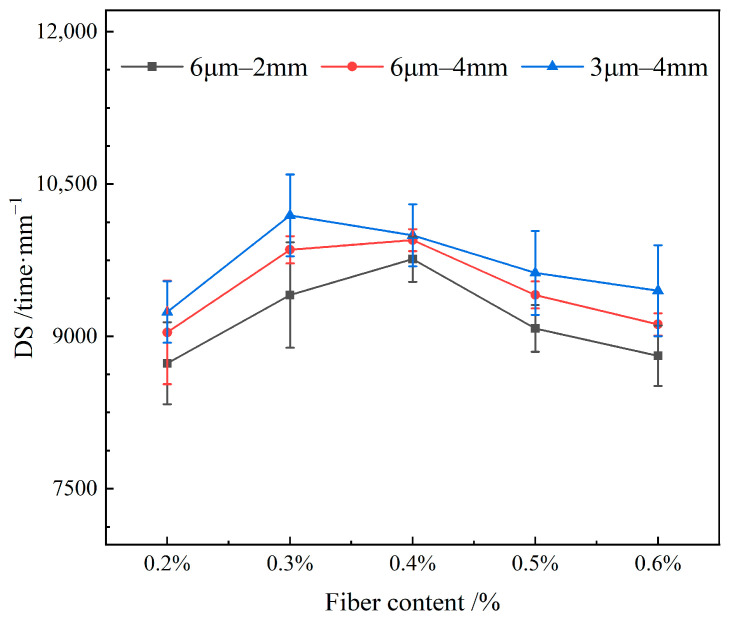
Effect of fiber content on high-temperature stability.

Figure 13 depicts the influence of varying the content of FBF on the high-temperature stability in RAM-SMA-13.

Figure 13 demonstrates that the dynamic stability of RAM-SMA-13 initially increases and subsequently decreases as the fiber content rises. Specifically, for FBF with dimensions of 6 μm in diameter and lengths of 4 mm and 2 mm, the peak dynamic stability is observed at a fiber content of 0.4%. Conversely, FBF with a diameter of 3 μm and a length of 4 mm achieves optimal high-temperature stability at a fiber content of 0.3%. At lower fiber concentrations, the fibers are uniformly dispersed within the mixture, forming an interconnected network that restricts the free movement of asphalt, thereby enhancing its performance. However, when the fiber content surpasses the optimal threshold, the uniformity of fiber distribution diminishes, weakening the reinforcing effect and leading to a decline in high-temperature stability.

#### 4.3.3. Low-Temperature Crack Resistance

**Figure 14:**
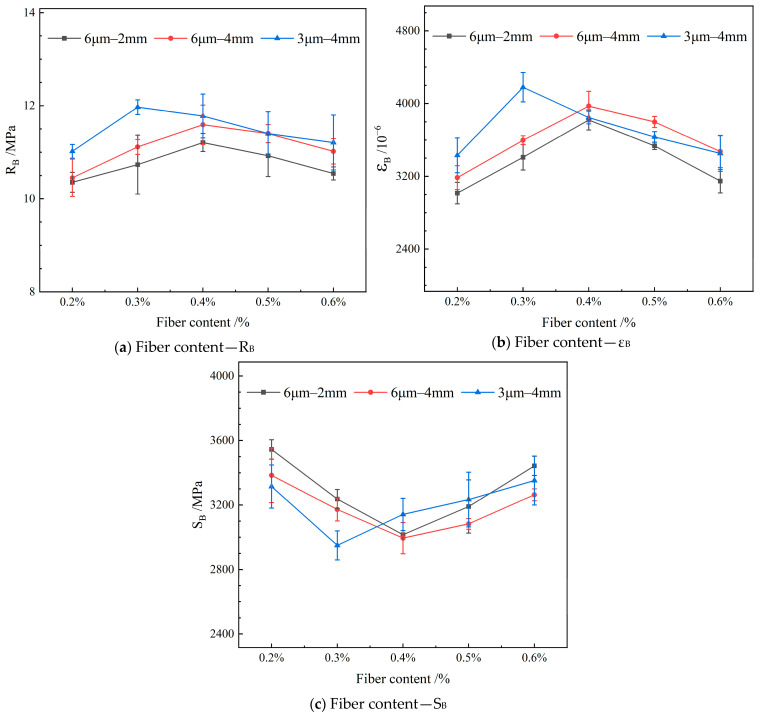
Effect of fiber content on low-temperature crack resistance.

Figure 14 depicts the influence of varying the length of the FBF on the low-temperature crack resistance in RAM-SMA-13.

Figure 14 demonstrates that the flexural tensile strength and flexural tensile strain of the mixture initially increase as the FBF content rises, followed by a subsequent decrease. In contrast, the flexural modulus displays an opposite trend, decreasing at first and then increasing. Specifically, for FBFs with a diameter of 6 μm and lengths of 4 mm and 2 mm, the optimal resistance to low-temperature cracking is achieved at a fiber content of 0.4%. Conversely, FBFs with a diameter of 3 μm and a length of 4 mm attains maximum low-temperature crack resistance at a fiber content of 0.3%. The incorporation of fibers hinders crack propagation through a bridging mechanism, and their relatively high elastic modulus and tensile strength enhance the composite’s toughness, thereby improving resistance to low-temperature cracking. However, excessive fiber content can impede adequate asphalt coating and bonding, leading to fiber agglomeration. These aggregated zones are susceptible to crack initiation and propagation under low-temperature loading conditions, ultimately diminishing the reinforcing effectiveness of FBF in enhancing the crack resistance of RAM.

Li et al. examined the influence of basalt fibers on the low-temperature performance of asphalt mixtures in cold climates, reporting that within a fiber content range of 0.2% to 0.5%, bending stress, bending strain, and strain energy density all increase [25]. Unlike the initial rise followed by a decline observed in the present study, their performance curves exhibit a more gradual variation. This difference is attributed to the presence of recycled aggregates and aged asphalt in RAM-SMA-13, which are more sensitive to the adsorption and network-forming properties of the fibers.

#### 4.3.4. Water Stability

**Figure 15:**
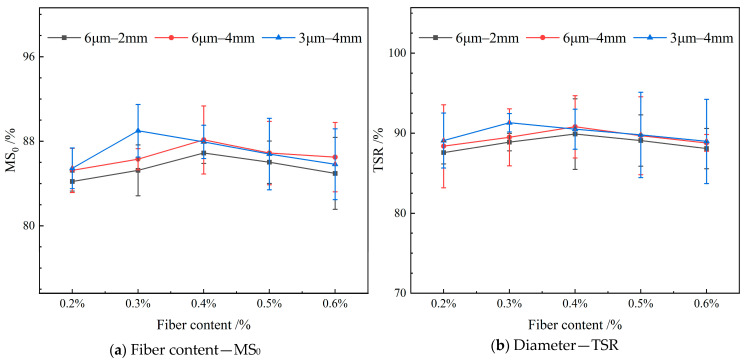
Effect of fiber content on water stability.

Figure 15 depicts the influence of varying the content of FBF on the water stability in RAM-SMA-13.

As illustrated in Figure 15, both the residual stability and freeze–thaw splitting strength of RAM-SMA-13 initially increase with the incorporation of fiber content, reaching a maximum before subsequently declining. Specifically, FBFs with dimensions of 6 μm in diameter and lengths of 4 mm and 2 mm demonstrate optimal water stability at a fiber content of 0.4%. Conversely, FBFs with a diameter of 3 μm and a length of 4 mm, as well as those measuring 6 μm by 4 mm, achieve peak water stability at a fiber content of 0.3%. The large surface area of these fibers facilitates the adsorption of free asphalt, resulting in the formation of a more robust structural asphalt film at the asphalt–aggregate interface. This enhanced interfacial adhesion improves bonding strength, thereby increasing the mixture’s stability. However, excessive fiber content negatively impacts fiber dispersion within the mixture, disrupts the microstructure, elevates porosity, and creates channels for water ingress, ultimately reducing the positive effects on water stability. In contrast, the water stability of RAM-SMA-13 appears largely unaffected by variations in FBF content.

In summary, the optimal flocculent basalt fiber content for improving the road performance of RAM-SMA-13 is identified as 0.4% for fibers with a diameter of 6 μm and lengths of either 4 mm or 2 mm, and 0.3% for fibers with a diameter of 3 μm and a length of 4 mm.

It is important to note that these findings are based on the idealized assumption of uniform fiber dispersion. While such uniformity can be readily achieved under controlled laboratory conditions, fiber agglomeration remains a common issue in practical large-scale engineering applications. Therefore, rigorous control of the mixing process is essential to ensure optimal fiber distribution during implementation.

## 5. Quantitative Evaluation of the Geometric Properties of Flocculent Basalt Fibers

Random Forest (RF), an ensemble learning method consisting of multiple decision trees, constitutes a robust approach for evaluating variable importance and conducting weight analysis. This technique effectively captures nonlinear relationships between fiber parameters—such as diameter, length, and content—and road performance without necessitating prior assumptions. Furthermore, RF outperforms traditional regression methods in modeling complex interactions among multiple variables, while simultaneously delivering a high predictive accuracy and interpretable assessments of feature significance. Subsequently, weight analysis is conducted to identify the principal factors influencing the OAR and the performance characteristics of RAM-SMA-13. In this study, the weight analysis derived from the RF model reflects the relative contribution of each factor to the model’s predictive capability, rather than representing the absolute magnitude of the factor weights.

### 5.1. Weight Analysis of the Asphalt–Aggregate Ratio

The input features selected for this study include fiber size characteristics, namely diameter and length, along with content, while the outcome variable is the OAR of RAM-SMA-13. The dataset is partitioned such that 20% constitutes the test set, with the random state fixed at 42 to ensure reproducibility and mitigate variability arising from random data splits. A 5-fold cross-validation approach is employed to stratify the dataset, facilitating multiple iterations of training and validation to reduce the impact of random small sample divisions on model performance. The number of decision trees is varied from 1 to 200 in increments of 25, with the optimal number determined once model accuracy reaches stability, accommodating the constraints of the limited sample size. Furthermore, the model’s average error is assessed, and the final ranking of the factors influencing the outcome, based on their relative importance, is depicted in Figure 8.

Table 8 reveals several key insights:

(1) Fiber content emerges as the most critical factor, as indicated by its highest weighting in the analysis, whereas fiber diameter and length exhibit substantially lower significance.
(2) Fiber content significantly affects the OAR within the mixture. Notably, increasing fiber content more effectively enhances the specific surface area of fibers in RAM-SMA-13 compared to modifications in fiber size parameters. The addition of fibers modifies the bonding characteristics of the mixture, thereby necessitating an increase in asphalt content to optimize the OAR and achieve superior performance.
(3) The impact of fiber size characteristics is relatively minor, likely due to their limited influence on the overall fiber performance within the mixture and on asphalt demand. Moreover, the homogeneous dispersion of fibers throughout the mixture, combined with appropriate adjustments in fiber content, further mitigates these effects.

### 5.2. Weight Analysis of the Road Performance

Using fiber size characteristics and content as the primary input variables, and the road performance of RAM-SMA-13 as the response variable, a two-stage hyperparameter optimization approach is employed to enhance the model’s predictive accuracy. Initially, Randomized Search CV is utilized to explore and refine the range of decision tree numbers (from 50 to 200) and maximum depths (from 10 to 40) in a gradient fashion, aiming to identify the most effective hyperparameter combination. Subsequently, Grid Search CV conducted an exhaustive search across all parameters to determine the global optimal set. The model is then trained using these optimal parameters, and finally, an importance ranking of the influencing factors is generated.

#### 5.2.1. Mechanical Properties

Table 9 displays the results pertaining to compressive strength (R_C_) and splitting tensile strength (R_T_).

As illustrated in Table 9, the mechanical properties of RAM-SMA-13 are significantly influenced by the fiber content and diameter, while the effect of fiber length is relatively limited. The fiber content and diameter of the FBF play a crucial role in determining the dispersion and spatial distribution of fibers within the composite. These parameters are more easily regulated during processing and fabrication, enabling the achievement of a consistent fiber proportion and uniform dispersion. In contrast, increasing fiber length may introduce processing and construction challenges, such as heterogeneous mixing, which can hinder improvements in strength performance and consequently reduce its overall impact on the mechanical properties.

#### 5.2.2. High-Temperature Stability

Table 10 presents the findings related to dynamic stability (DS).

As illustrated in Table 10, the content and diameter of FBF exert a more significant influence on high-temperature stability than fiber length, which demonstrates a relatively minor effect. The FBF content and diameter are essential parameters for establishing an effective fiber spatial network and strengthening the interfacial bonding with the asphalt binder. While fiber length plays a role in enhancing dynamic stability mainly by controlling crack propagation and increasing toughness, the overall dynamic stability is largely determined by the fiber network configuration and the interfacial adhesion between the fibers and asphalt. Therefore, the effect of FBF length on dynamic stability is comparatively limited.

#### 5.2.3. Low-Temperature Crack Resistance

Table 11 displays the results pertaining to flexural tensile strength (R_B_), flexural modulus (S_B_), and flexural tensile strain (ε_B_).

As illustrated in Table 11, the content and diameter of the FBF play a critical role in enhancing resistance to low-temperature cracking, whereas the effect of fiber length is comparatively minor. Although variations in FBF length can moderately improve the composite’s toughness, the content and diameter of the FBF have a more pronounced impact on the uniform dispersion within the asphalt binder and the development of its structural framework. The establishment of a robust three-dimensional network facilitates more efficient stress distribution, thereby enhancing both flexural tensile strength and flexural modulus. Moreover, the content and diameter of the FBF represent more readily controllable parameters that promote homogeneous fiber dispersion, in contrast to longer fibers, which may result in uneven distribution and adversely affect overall composite performance. Therefore, adjustments in FBF content and diameter yield more substantial improvements in the mechanical properties of the composite.

#### 5.2.4. Water Stability

Table 12 presents the findings related to residual stability (MS_0_) and the freeze–thaw splitting strength ratio (TSR).

As illustrated in Table 12, both the content and diameter of the FBF exert a significant influence on water stability, whereas the length of the FBF has a comparatively minor effect. The fiber content predominantly affects the network structure and the bonding strength among fibers, thereby markedly enhancing the overall properties of RAM-SMA-13. While an increase in FBF length contributes to improved toughness, its impact on the formation of a robust fiber network is notably less substantial compared to the other two factors. Furthermore, the use of longer fibers may lead to uneven dispersion within RAM-SMA-13, potentially diminishing uniformity and consequently limiting the extent of performance enhancement.

## 6. Conclusions

This study investigates the SMA-13 recycled asphalt mixture (RAM-SMA-13), aiming to analyze the combined effects of flocculent basalt fiber (FBF) size parameters (diameter and length) and fiber content on key performance indexes. Additionally, a random forest approach is employed to evaluate the relative significance of FBF size parameters as well as content in relation to these performance indexes. The principal findings are summarized as follows:

(1) At equal fiber content, decreasing fiber diameter and increasing fiber length raise the optimal asphalt–aggregate ratio (OAR). Moreover, the influence of fiber diameter on the OAR is more pronounced than that of fiber length. This is because finer fibers have a larger specific surface area, enhancing the adsorption capacity of asphalt mastic, while longer fibers form a denser spatial network to trap the asphalt.
(2) The FBF size parameters and content collectively influence the overall performance of RAM-SMA-13 by optimizing the spatial skeleton structure and enhancing the bonding between asphalt and aggregate. Reducing the fiber diameter while increasing its length can simultaneously improve mechanical properties, high-temperature stability, resistance to low-temperature cracking, and water stability. Regarding the effect of FBF content, all performance indexes initially improve but then decline as FBF content increases. The optimal content thresholds are identified as 0.4%; beyond this level, fiber agglomeration occurs, compromising the mixture’s uniformity and diminishing its overall performance.
(3) The weight of FBF size parameters and content on the OAR and overall performance has been analyzed. Fiber content has the most significant impact, followed by fiber diameter, while fiber length has the least influence. This established hierarchy provides a theoretical basis for prioritizing the adjustment of FBF size parameters in engineering practice.
(4) The optimal fiber content for enhancing the road performance of RAM-SMA-13 has been identified as 0.4% for fibers with a diameter of 6 µm (regardless of fiber length in this study) and 0.3% for fibers with a diameter of 3 µm and a length of 4 mm.
(5) Compared to previous research, this study offers two primary contributions. First, it systematically elucidates the combined effects of FBF size parameters and content on the performance of RAM-SMA-13. Second, it quantitatively establishes the relative influence of FBF content, diameter, and length, and identifies the optimal parameter combinations. These findings provide a theoretical foundation for the precise application of FBF in recycled asphalt pavement engineering.
(6) Regarding the limitations of this study, a broader spectrum of FBF specification combinations will be systematically evaluated to assess the effect of FBF size parameters on the road performance of RMA in future studies. Simultaneously, microscopic analyses, including SEM, XRD, and TGA, will be performed to elucidate the interaction mechanisms between the FBF and the RMA.

## Figures and Tables

**Table 1:** Technical properties of modified asphalt.

Technical Indexes		Experimental Results	Standards
25 °C penetration/0.1 mm penetration		51	40–60
5 °C ductility/cm		60.6	≥20
Softening point/°C		81.9	≥60
Dynamic viscosity at 135 °C/Pa·s		2.001	≤3.0
Elastic recovery at 25 °C/%		89	≥75
Rotating film aging test	Mass loss/%	−0.33	≤±1.0
	5 °C ductility/cm	53	≥15
	25 °C residual penetration ratio/%	71.1	≥65

**Table 2:** Technical properties of coarse aggregates.

Technical Indexes	Experimental Results		Standards
	9.5–13.2 mm	4.75–9.5 mm	
Apparent relative density	2.766	2.769	≥2.6
Needle and flake content/%	6.9	7.8	≤15
Water absorption rate/%	0.68	0.53	≤2.0
Crush value/%	13.7		≤26
Wear value/%	18.9		≤28
Durability/%	7.7		≤12

**Table 3:** Technical properties of fine aggregates.

Technical Indexes	Experimental Results	Standards
Apparent relative density	2.671	≥2.5
Firmness/%	6.2	≤12
Methylene blue value/g·kg	2.1	≤25
Angular/s	39.9	≥30

**Table 4:** Technical properties of mineral powder.

Technical Indexes		Experimental Results	Standards
Apparent relative density		2.666	≥2.5
Moisture content/%		0.3	≤1
Size range/%	<0.6 mm	100	100
	<0.15 mm	94.3	90–100
	<0.075 mm	91.0	75–100
Hydrophilic coefficient		0.6	<1
Plasticity index/%		2.7	<4

**Table 5:** Technical properties of recycled aggregates.

Technical Indexes	Experimental Results		Standards
	0–8 mm	8–12 mm	
Apparent relative density	2.597	2.689	≥2.5 (0–8 mm) ≥2.6 (8–12 mm)
Hair volume relative density	-	2.771	-
Mud content/%	2.6	-	≤3
Sand equivalent/%	72	-	≥60
Water absorption rate/%	-	0.97	≤2.0
Needle and flake content/%	-	11.1	≤15
Crushing value/%	-	13.7	≤26
<0.075 particles/%	-	0.39	≤1
Los Angeles abrasion loss/%	-	26	≤28

**Table 6:** Technical properties of flocculent fiber.

Technical Indexes	Experimental Results		
Diameter/μm	6	3	6
Length/mm	4	4	2
Density/g/cm^3^	2.697	2.597	2.691
Melting point/°C	1250	1250	1250
Moisture content/%	0.12	0.13	0.16
Tensile strength/MPa	1260	1380	1260
Elastic modulus/GPa	80	40	80
Elongation at break/%	3.3	2.7	3.3

**Table 7:** Aggregate gradation.

Mass Percentage/% Passing Through the Following Sieve Apertures/mm								
13.2	9.5	4.75	2.36	1.18	0.6	0.3	0.15	0.075
100	55	36	25	19	13	11	8	6

**Table 8:** S-SMA-Feature Importance ranking table.

No.	Influencing Factors	Weighted Values
1	Content	0.8994
2	Diameter	0.0654
3	Length	0.0352

**Table 9:** Weight table of factors affecting mechanical properties.

Influencing Factors	R_C_ Weight Value	R_T_ Weight Value
Length	0.166	0.029
Diameter	0.392	0.059
Content	0.442	0.912

**Table 10:** Weight table of factors affecting high-temperature stability.

Influencing Factors	DS Weight Value
Length	0.113
Diameter	0.259
Content	0.628

**Table 11:** Weight table of factors affecting low-temperature crack resistance.

Influencing Factors	R_B_ Weight Value	ε_B_ Weight Value	S_B_ Weight Value
Length	0.120	0.122	0.146
Diameter	0.374	0.193	0.110
Content	0.506	0.685	0.744

**Table 12:** Weight table of factors affecting water stability.

Influencing Factors	MS_0_ Weight Values	TSR Weight Values
Length	0.173	0.122
Diameter	0.145	0.263
Content	0.682	0.615

## Data Availability

The original contributions presented in this study are included in the article. Further inquiries can be directed to the corresponding authors.
